# Atrophic gastritis rather than Helicobacter pylori infection can be an independent risk factor of colorectal polyps: a retrospective study in China

**DOI:** 10.1186/s12876-023-02764-w

**Published:** 2023-06-19

**Authors:** Zhang Shao-hua, Ren Lin-lin, Su Shen, Tang Yun-he, Tian Zi-bin, Liu Yi, Mao Tao

**Affiliations:** Department of Gastroenterology, the Affiliated Hospital of Qingdao University, No. 16, Jiangsu Road, Qingdao, 266003 Shandong Province China

**Keywords:** Atrophic gastritis, Helicobacter pylori, Colorectal polyp, Retrospective study

## Abstract

**Background:**

Colonoscopy is considered the most effective screening method for colorectal polyps. However, the longevity and complexity of the procedure makes it less desirable to screen for colorectal polyps in the general population. Therefore, it is essential to identify other independent risk factors. In this study, we explored the link between Hp infection, atrophic gastritis, and colorectal polyps to identify a new potential risk factors of colorectal polyps.

**Methods:**

In this study, atrophic gastritis and intestinal polyps were diagnosed by endoscopy and pathology. All the 792 patients in this retrospective study were divided into sub-groups based on the presence of colorectal polyps. The correlation between polyps and atrophic gastritis was analyzed using the chi-square test and Kruskal-Wallis test. The receiver operating characteristic (ROC) curve was used to compare the predictive value for colorectal polyps between Hp infection and atrophic gastritis. Binary logistic regression was utilized to identify independent risk factors for colorectal polyps.

**Results:**

Patients with colorectal polyps were primarily male with advanced age, and the number of patients with colorectal polyps had a higher association with smoking, alcohol drinking, and Hp infection than the control group. A positive correlation between the number of colorectal polyps and the severity of atrophic gastritis was observed. ROC analysis showed that atrophic gastritis was a better risk factors for colorectal polyps. Multivariate analysis identified atrophic gastritis as an independent risk factor for colorectal polyps (OR 2.294; 95% CI 1.597–3.296).

**Conclusions:**

Atrophic gastritis confirmed could be an independent risk factors for colorectal polyps.

## Introduction

Colorectal cancer is the third most common cancer and the fifth leading cause of cancer-related deaths worldwide [1]. Colorectal polyps, especially adenomatous polyps, are considered pre-cancerous lesions caused by numerous factors such as smoking, age, and excessive red meat intake [2]. Colonoscopy is the gold standard for detecting pre-cancerous lesions such as colorectal polyps. Due to operational and preparation complexity, and the inherent risks involved with the procedure, especially in regions with insufficient medical resources, it is challenging to use colonoscopy for mass screening [3]. Hence a more convenient, efficient way to identify high-risk patients is necessary, which can help physicians to decide whether a patient should be subjected to an invasive procedure like a colonoscopy.

Helicobacter pylori (Hp) is recognized as a type I carcinogen by World Health Organization [4, 5] and plays an important role in the development of gastric cancer [6–8]. Long-term Hp infection leads to atrophic gastritis and intestinal epithelial metaplasia, which eventually may progress into gastric cancer [9]. In addition to gastric cancer, Hp infection is associated with various digestive diseases, such as colorectal cancer. Interestingly a report suggests an association between Hp infection and colorectal polyps [10]. Hp infection promotes the secretion of gastric acid and causes colorectal polyps [11, 12]. Whether the correlation between Hp infection and colorectal polyps is mediated by Hp-associated atrophic gastritis is yet to be established. Therefore, this retrospective study was conducted to elucidate the relationship between atrophic gastritis, Hp infection, and colorectal polyps to provide new insight into screening strategies for colorectal polyps.

## Methods

### Patients

Patients who underwent their first gastroscopy combined with colonoscopy at the Affiliated Hospital of Qingdao University from January 2021 to March 2022 were enrolled for the study. Patients diagnosed with gastric cancer, colon cancer, ulcerative colitis, Crohn’s disease, MALT lymphoma, gastric submucosal masses, and familial adenomatous polyposis were excluded from the study. Further patients who underwent previous gastrointestinal resection were also excluded from the study.

Patients with colorectal polyps were categorized as the polyp group, while patients with no detectable colorectal polyps were classified as the control group.

### Data collection

Gastroscopy biopsy specimens and carbon 13 urea breath test was used to detect Hp infection in the patients. Patients confirmed positive for either of these tests were considered positive for Hp infection. Information on previous Hp infection and treatment plans was obtained from all the patients through inquiry and medical records. Two experienced physicians graded the degree of gastric atrophy in patients using the Kimura-Takemoto classification [13] and gastroscopy, and the atrophic gastritis was classified into open and closed types. If the atrophic region was just limited to the lesser curvature of the stomach was regarded as a closed type (C type) and was divided into three grades (C-1, C-2, C-3). If the atrophic region extended to the greater curvature side was regarded as an open type and was classified into three grades (O-1, O-2, O-3). All polyps found were excised carefully and sectioned for pathological evaluation. Hematoxylin and eosin staining was performed on the sections for diagnosis. Polyps were classified into neoplastic polyps, which included tubular adenomas, tubular villous adenomas, choroidal and serrated adenomas. The second type was non-neoplastic polyps, which included hyperplastic polyps and inflammatory polyps. Polyp size was assessed per the Yamada criteria, and the number of polyps was classified as single or multiple.

### Statistical analysis

Quantitative data were expressed as mean value ± standard deviation (X ± S), while qualitative data was represented as n (%). Differences between the two sets of qualitative data were compared using the Chi-square test or Fisher’s exact test. The quantitative data were calculated using the parametric test like student’s t-test, and non-parametric tests like Kruskal-Wallis test. The univariate analysis compared the clinicopathological features between the two groups and was calculated using the Chi-square test, Fisher’s exact test, student’s t-test, and Kruskal-Wallis test. Multivariate regression analysis was performed on variables with P < 0.05 and was calculated using the odds ratio (OR) and a 95% confidence interval (95% CI). A binary logistic regression analysis was used to identify independent risk factors for colorectal polyps. Receiver operating characteristic (ROC) curve was used to compare the predictability of colorectal polyps by Hp infection and atrophic gastritis. P < 0 0.05 was considered as statistically significant. The data analysis was performed using SPSS version 26 (SPSS Inc., Chicago, IL, USA).

## Results

### Demographics of study patients

A total of 792 patients were included in the study. The mean age of patients was 54.34 ± 10.92 years, of whom 381 (48.1%) were males, and 411 (51.9%) were females. Colorectal polyps were diagnosed in 385 (48.7%) patients, while a total of 332 patients (41.9%) were diagnosed with atrophic gastritis. Table 1 shows the clinical characteristics of all the patients. Significant differences in gender, mean age, body mass index (BMI), alcohol drinking habits, smoking habits, diabetes, atrophic gastritis, and present Hp infection status were observed between the colorectal polyp and the control group. P < 0.05 for all the characteristics mentioned above, indicating statistically significant differences.

**Table 1:** Baseline characteristics of the 792 subjects according to colorectal polyps status

	Overall (n = 792)	polyp group(n = 385)	control group (n = 407)		P value
Age	54.34 ± 10.92	56.60 ± 9.15	52.21 ± 11.99	<0.05	
Gender				<0.05	
Male	381	219	162		
Female	411	166	245		
BMI	25.03 ± 3.46	25.51 ± 3.30	24.58 ± 3.55	<0.05	
Atrophy				<0.05	
Positive	332	209(54.3%)	123(30.2%)		
Negative	460	176	284		
Smoking habits	102	69	33	<0.05	
Alcohol drinking	102	66	36	<0.05	
Hypertension				0.07	
Positive	251	134	117		
Negative	541	251	290		
Diabetes				<0.05	
Positive	127	72	55		
Negative	665	313	352		
Hp infection				<0.05	
Positive	312	168	144		
Negative	480	217	263		

### The relationship between atrophic gastritis and the incidences of colorectal polyps

The incidence of colorectal polyps in patients with atrophic and non-atrophic gastritis was analyzed. The number of colorectal polyps in patients with atrophic gastritis was significantly higher than in non-atrophic gastritis patients (P < 0.05) and significant difference was observed in the diagnosis of adenoma and the polyp diameter between the two groups (Table 2).

**Table 2:** Comparison of polyps features of patients according to types of gastritis

	Atrophic gastritis (n = 332)	Non-atrophic gastritis(n = 459)		P value
Pathology				0.048
Adenoma	153		110	
hyperplastic polyp	31		34	
inflammatory polyp	26		35	
Average number	1.74 ± 2.56		0.89 ± 2.05	<0.05
Average size	6.13 ± 3.63		6.39 ± 5.82	0.48

### Correlation between the severity of atrophic gastritis and colorectal polyps

To understand the association between atrophic gastritis and colorectal polyps, the atrophic gastritis patients were further classified based on the severity of atrophy, and Kruskal-Wallis test was performed (Table 3). O-2 and O-3 type gastritis were excluded as only one patient was graded as O-1 in the current study. As shown in Table 3, the incidence and number of polyps positively correlated with the severity of atrophy (P < 0.05).

**Table 3:** Comparison of polyps features of patients according to atrophic gastritis grade

		C-1 (n = 169)	C-2 (n = 115)	C-3 (n = 34)	O-1 (n = 13)	P value
Polyp						0.018
Positive		95	75	27	11	
Negative		74	40	7	2	
Average number of polyps		1.34 ± 1.77	1.81 ± 2.65	2.94 ± 4.37	3.15 ± 2.97	0.006

### The relationship between atrophic gastritis and colorectal polyps

Previous studies have shown an association between Hp infection and colorectal polyps. This association focused on current Hp infections, whereas patients with the previous infection mainly were neglected. To further investigate the association between Hp infection and colorectal polyps, the patients were divided into three groups: patients with current Hp infection, patients with previous Hp infection, and Hp negative patients (Table 4).

Logistic regression analysis showed that gender, mean age, body mass index (BMI), alcohol drinking habits, smoking habits, diabetes, atrophic gastritis, and current Hp infection were associated with colorectal polyps. Multivariate analysis (Table 4) revealed that age (P<0.05, OR = 1.032), gender (P < 0.05, OR = 1.545) and atrophic gastritis (P < 0 0.05, OR = 2.294) were independent risk factors for colorectal polyps.

**Table 4:** Multivariate logistic regression analyses of risk factors of colorectal polyps

	Colorectal polyps	
	Multivariate analysis	
	OR (95%CI)	P value
Age	1.032(1.017–1.048)	<0.05
Atrophic gastritis	2.294(1.597–3.296)	<0.05
Gender	1.545(1.108–2.155)	<0.05
BMI	1.045(0.999–1.093)	0.057
Diabetes	1.090(0.722–1.645)	0.682
Smoking habits	1.516(0.826–2.781)	0.179
Alcohol drinking	1.166(0.638–2.130)	0.617
previous Hp infection	0.965(0.585–1.592)	0.890
current Hp infection	1.392(0.994–1.951)	0.054

### The predictive value of atrophic gastritis for colorectal polyps

The area under the curve (AUC) for the ROC curve analysis was performed to check the predictive value of atrophic gastritis and Hp infection for colorectal polyps. AUC for atrophic gastritis was 0.62 (P < 0.001) and for Hp infections was 0.57 (P < 0.001).

Although the value is low and should be combined with other indicators for a more accurate diagnosis, these results still reveal that atrophic gastritis was better than Hp infection in finding colorectal polyps in patients.

## Discussion

Previous studies have established that colorectal polyps, especially adenomatous polyps, are risk factors for colon cancer [14, 15]. Colonoscopy is the gold standard for detecting colorectal polyps before they become clinically symptomatic. However, several issues, including difficulties associated with the operations, the invasive nature of the procedure, and the steps involved in preparing the patients for a colonoscopy, are complex, which hampers the rampant use of colonoscopy for colorectal polyp screening in the general population [16]. Therefore, a simple, less invasive, and accessible strategy for screening colorectal polyps is required.

Several studies have shown that Hp infection is a risk factor for colorectal polyps [12, 17]. Hp causes hyperplasia of the intestinal mucosa by increasing the levels of gastrin, which can lead to the development of intestinal polyps [11, 18, 19]. However, some studies contradict this hypothesis [20]. Reports also suggest Hp directly induces atypical hyperplasia of the intestinal mucosa [21]via CagA, a Hp virulence factor [22]. In this study, we report Hp infection was a risk factor for colorectal polyps. To further evaluate the predictive value of Hp infection status for colorectal polyps, Hp infection was divided into previous Hp infection, current Hp infection, and Hp-negative group. Further analysis revealed that previous Hp infections (P = 0.054. OR = 0.721) and current Hp infections (P = 0.131. OR = 0.696) were not independent risk factors for colorectal polyps.

Hp infection leads to chronic inflammation, which causes damage to the gastric mucosa.

Previous studies have also demonstrated that atrophic gastritis with Hp infection is a risk factor for colorectal polyps [23]. However, in the current study, Hp infection was not an independent risk factor for colorectal polyps. It is important to note that the previous studies did not account for atrophic gastritis caused by Hp infection [12, 24]. Therefore, in this study, patients with atrophic gastritis were further categorized based on the severity of atrophic gastritis, and we discovered that the number of polyps increased with the severity of atrophic gastritis (P < 0.05), but the severity of atrophic gastritis did not make a difference to the size of polyps. These results confirm that atrophic gastritis could hint the development of colorectal polyps. Further ROC analysis confirmed that atrophic gastritis and different grades of atrophic gastritis could better predict the status of colorectal polyps than Hp infection.

The mechanism of atrophic gastritis leading to colorectal polyps is not yet fully understood [23]. Several studies show the relationship between intestinal microbiota and colorectal carcinogenesis [25, 26]. Wong et al. used feces of colorectal cancer patients to feed the mice, and the results show that the experimental group had a significantly higher proportion of heterogeneous hyperplasia and fleshy polyps in the colon compared to the control group [27]. Studies have also shown that atrophic gastritis could cause alterations in the intestinal flora [28], causing neoplastic transformation of the intestine by altering various signaling pathways [29]. Besides, the growth of certain bacteria in the intestine may increase the production of secondary bile acids, which may also contribute to developing colon polyps and cancer [30]. Furthermore, patients with atrophic gastritis are often more inclined to take proton pump inhibitors, which may significantly increase the incidence of intestinal adenomas [31]. What’s more, hypochlorhydria caused by atrophic gastritis may impede protein assimilation, giving rise to some unabsorbed metabolites and nutrients [32]. Hypochlorhydria leads to colorectal disorders, which will eventually result in colorectal polyps and even colorectal carcinogenesis [33].

However, the study has some limitations. This was a single-center and retrospective study with relatively small sample size, leading to biased results. Although atrophic gastritis was an independent risk factor of colorectal polyps and was better than Hp infection in hinting colorectal polyps, the sensitivity and specificity of atrophic gastritis were low, indicating that additional studies are needed to validate the results.

## Conclusion

Previous studies focus on the association between Hp infections and colorectal polyps [12, 17]. However, the relationship between atrophic gastritis and colorectal polyps has not yet been reported. In our study, we performed a subgroup analysis of different Hp infection statuses and found that atrophic gastritis was an independent risk factor for colorectal polyps. Thus, detecting atrophic gastritis using gastroscopy could effectively predict colonic polyps, indicating a brand-new screening strategy for colorectal polyps via gastroscopy.

## Data Availability

The datasets generated during and/or analysed during the current study are available from the corresponding author on reasonable request.
